# Supplementary material for: Zika M—A Potential Viroporin: Mutational Study and Drug Repurposing
Source: Biomedicines. 2022 Mar 10;10(3):641. doi: 10.3390/biomedicines10030641 (PMC8944957; doi:10.3390/biomedicines10030641)
Supplement: Supplementary file 1 [file biomedicines-10-00641-s001.zip › biomedicines-1599754-supplementary.pdf]

Zika M - A Potential Viroporin: Mutational Study and Drug Repurposing

Prabhat Pratap Singh Tomar, Miriam Krugliak, Anamika Singh, Isaiah T. Arkin

Supplementary figures

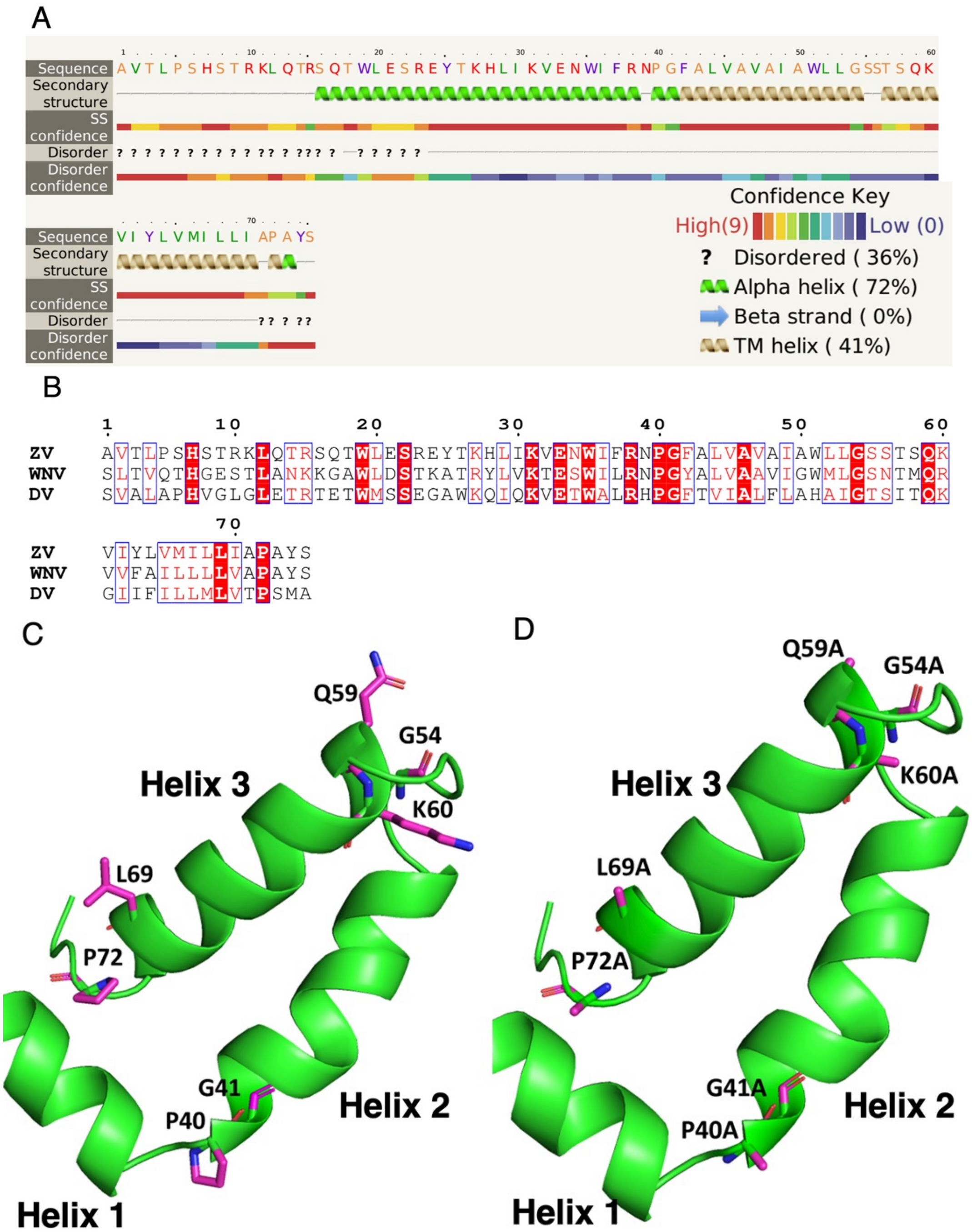

Supplementary figure 1. Structure of ZikV-M and comparison with other flavivirus viroporins. A. Sequence of matured ZikV-M, showing the secondary structure and Trans-membrane Domain (TMD). B. Sequence alignment of ZikV, WNV, and DenV proteins. The highlighted region in red showing conserved amino acids. C and D. Cartoon structure of the protein based on the available cryo-EM structures (5IRE and 6CO8). The visualization of the structure was done using the PyMOL Molecular Graphics System, Version 2.0 (Schrödinger, NY), showing original and substituted amino acids.

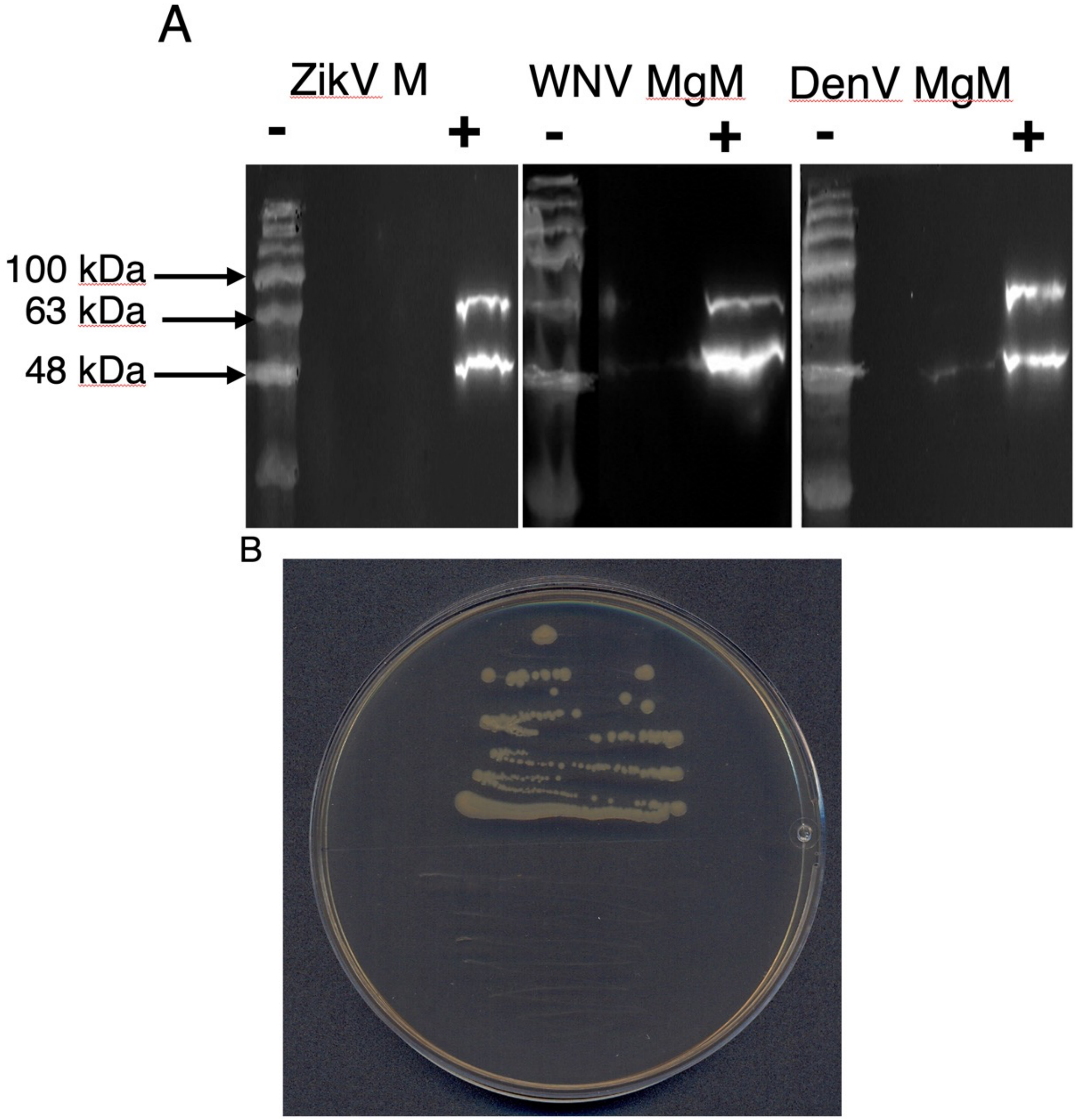

Supplementary figure 2. Expression and integration of ZikV-M. A. Western blot. Flavivirus chimeric constructs in DH10B cells were induced for 3 hours with 100 µM IPTG. Un-induced (without IPTG) cultures were taken as control. The presence of a band at around 50 kDa confirms protein expression. B. Maltose complementation assay. Bacteria that lack a maltose-binding protein (NT 326) were transformed with the pMAL-ZikV-M construct. NT 326 cells with pUC-19 vector were taken as control (botom. Cells were grown on M9 media with 1% of maltose at 37°C for 72 hours. The growth of bacteria that harbour ZikV M confirms the integration of protein in the membrane since it allows cells to use maltose as a carbon source.

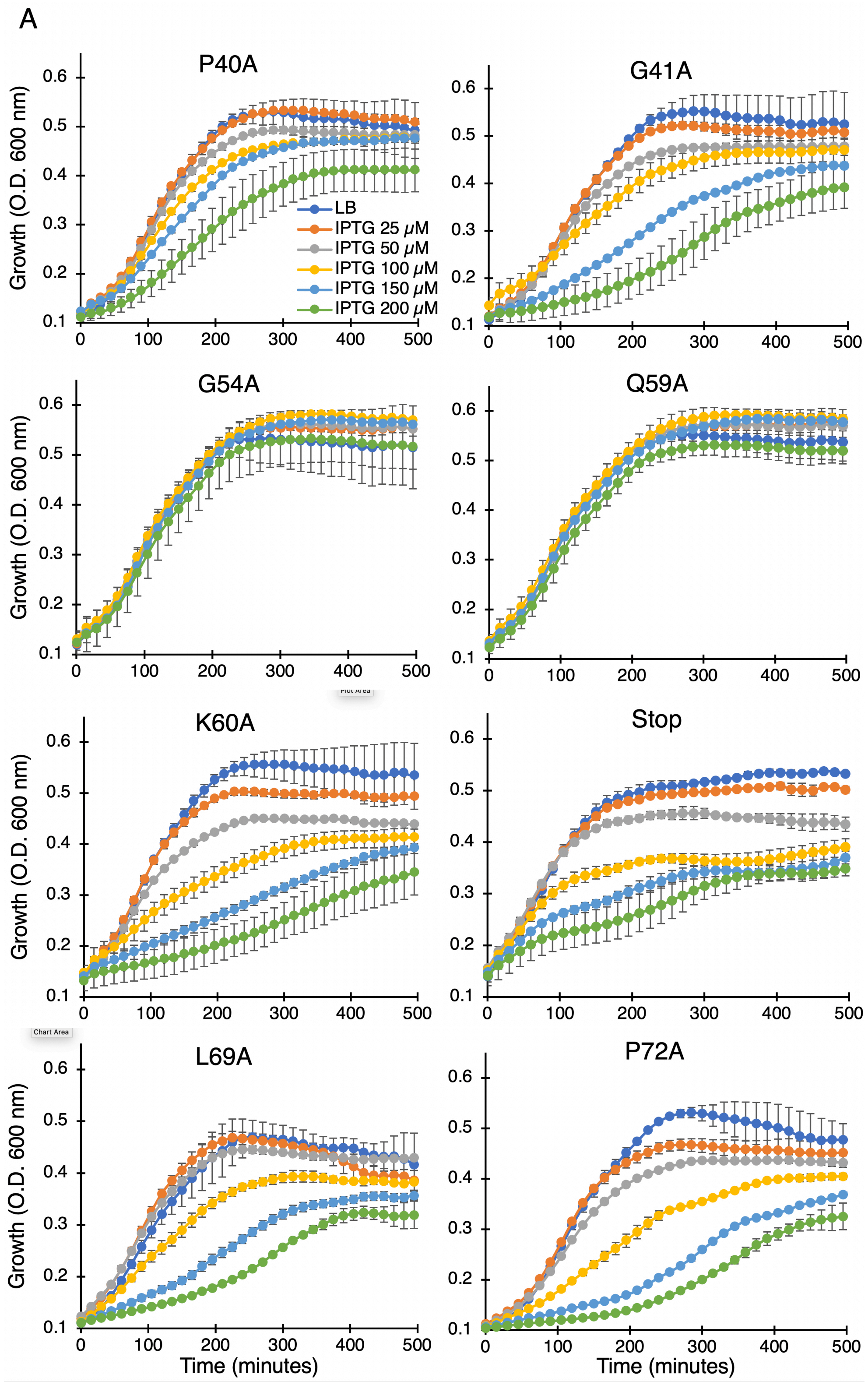

A
P40A
G41A
G54A
Q59A
K60A
Stop
L69A
P72A
Growth (O.D. 600 nm)
Time (minutes)
LB
IPTG 25 μM
IPTG 50 μM
IPTG 100 μM
IPTG 150 μM
IPTG 200 μM
Plot Area
Chart Area

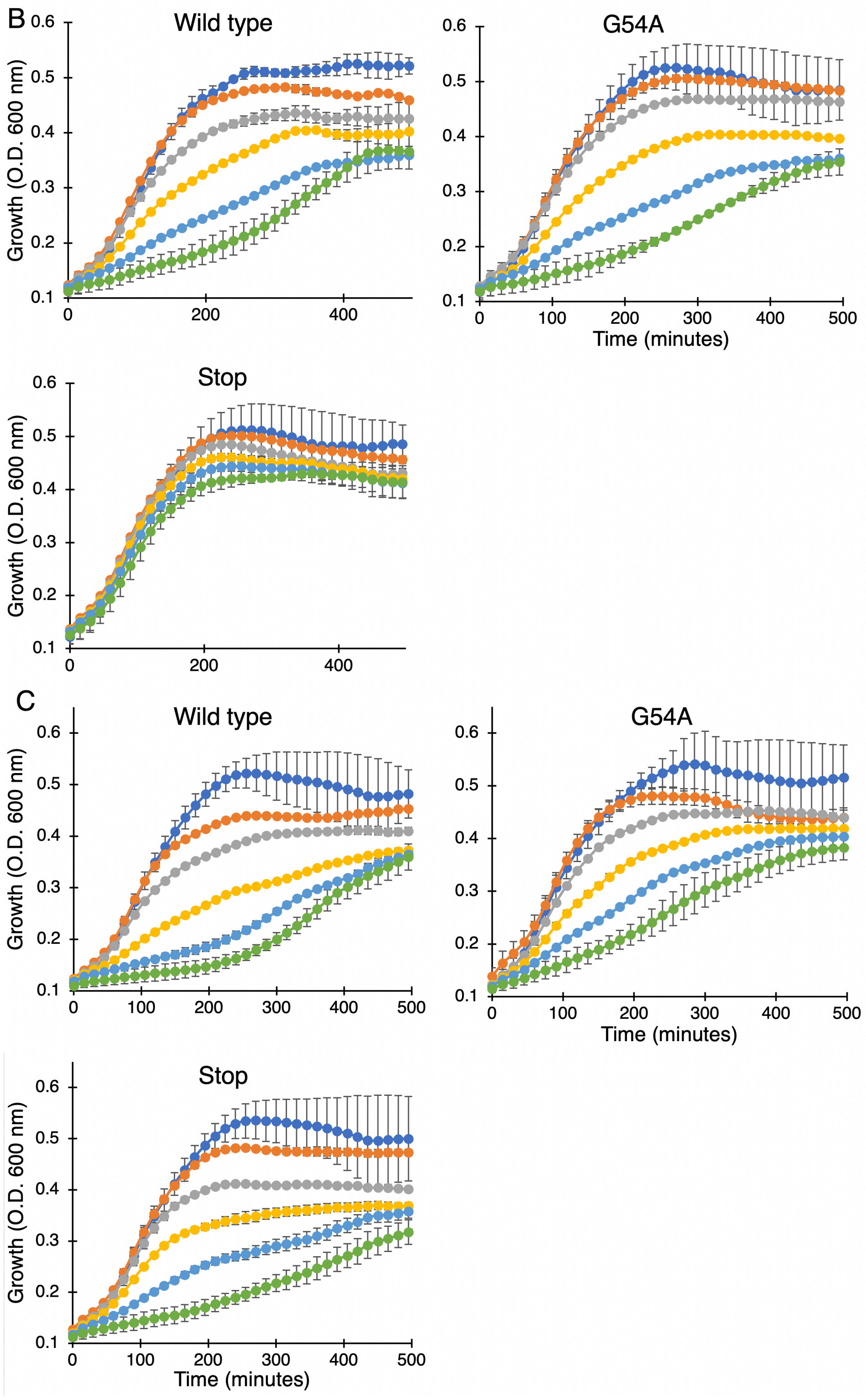

Supplementary figure 3. Negative genetic assay of flavivirus viroporins. Negative genetic assay of ZikV-M wild-type and mutants (A), WNV MgM wild-type and mutants (B) and DenV MgM wild-type and mutants (C). All figures depict growth curve as a function of the IPTG inducer. The different mutations are indicated.

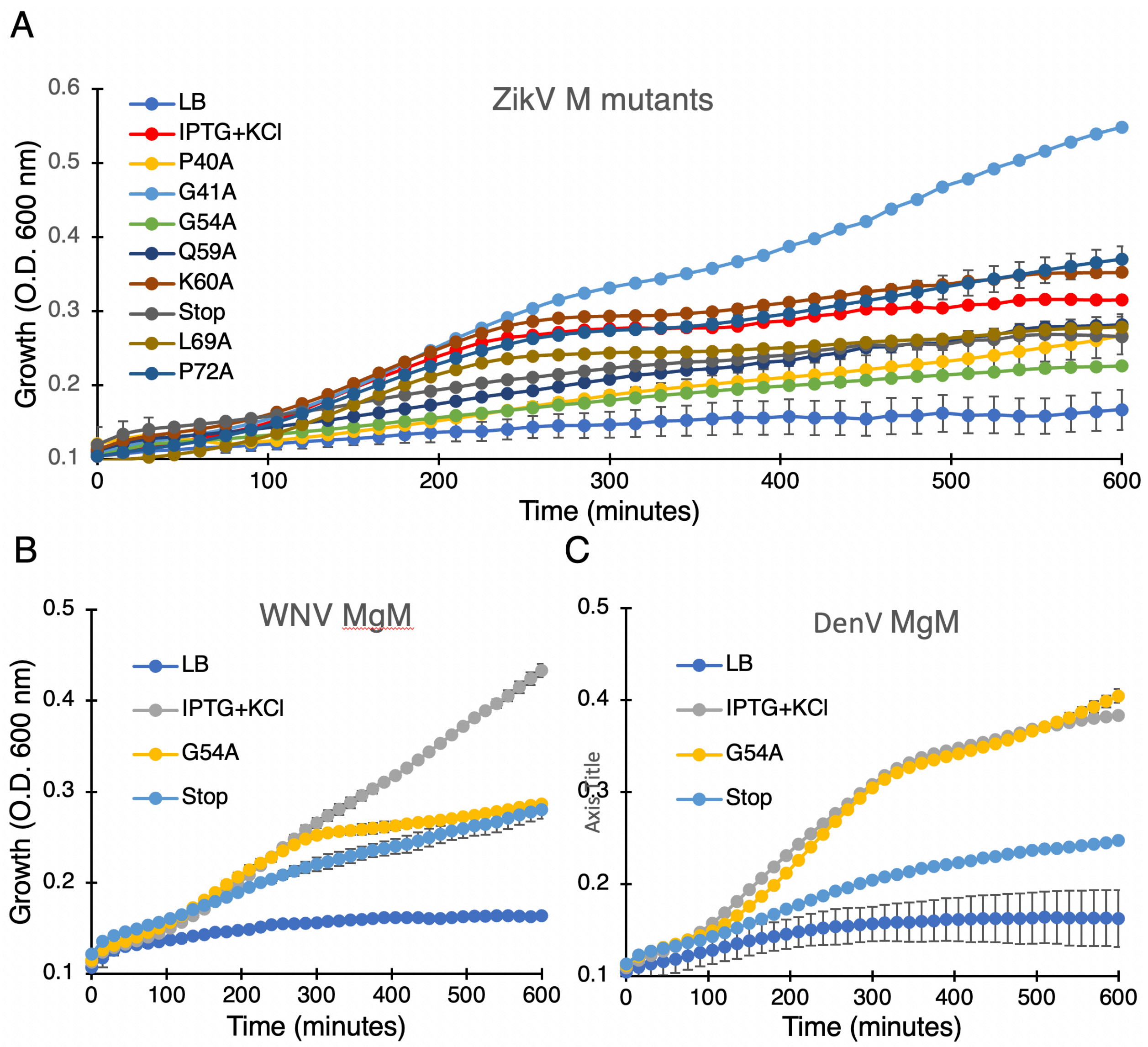

Supplementary figure 4. Positive genetic assay of flavivirus viroporins. Positive genetic assay of ZikV-M wild-type and mutants (A), WNV MgM wild-type and mutants (B) and DenV MgM wild-type and mutants (C). The different mutations are indicated.

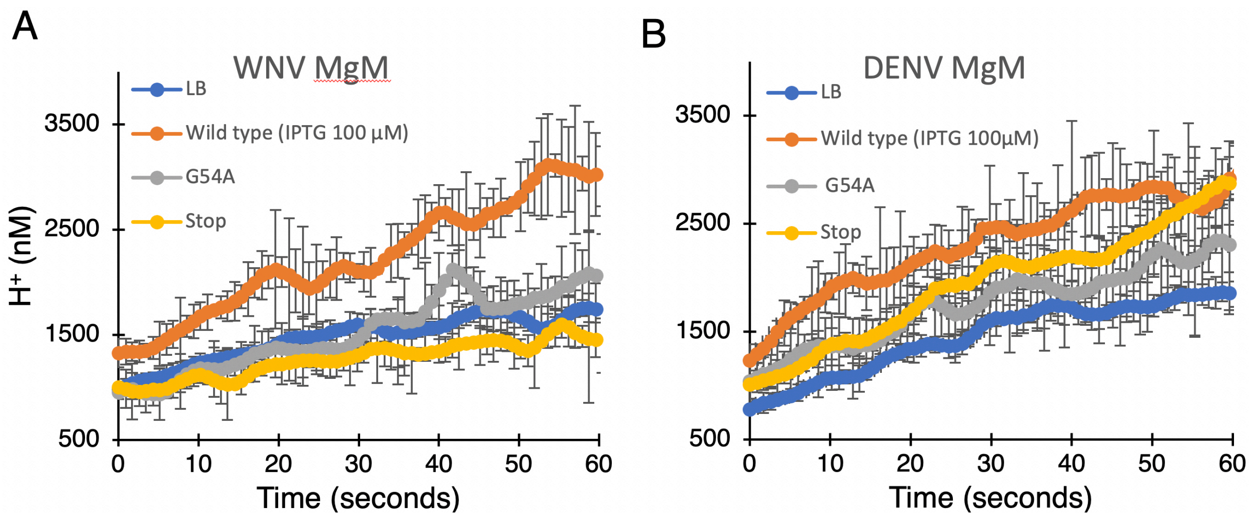

Supplementary figure 5. Proton flow assay of flavivirus viroporins. Proton flow assay of WNV MgM wild-type and mutants (A) and DenV MgM wild-type and mutants (B).
